# Supplementary figures and images for: A model of resource partitioning between foraging bees based on learning
Source: PLoS Comput Biol. 2021 Jul 28;17(7):e1009260. doi: 10.1371/journal.pcbi.1009260 (PMC8351995; doi:10.1371/journal.pcbi.1009260)

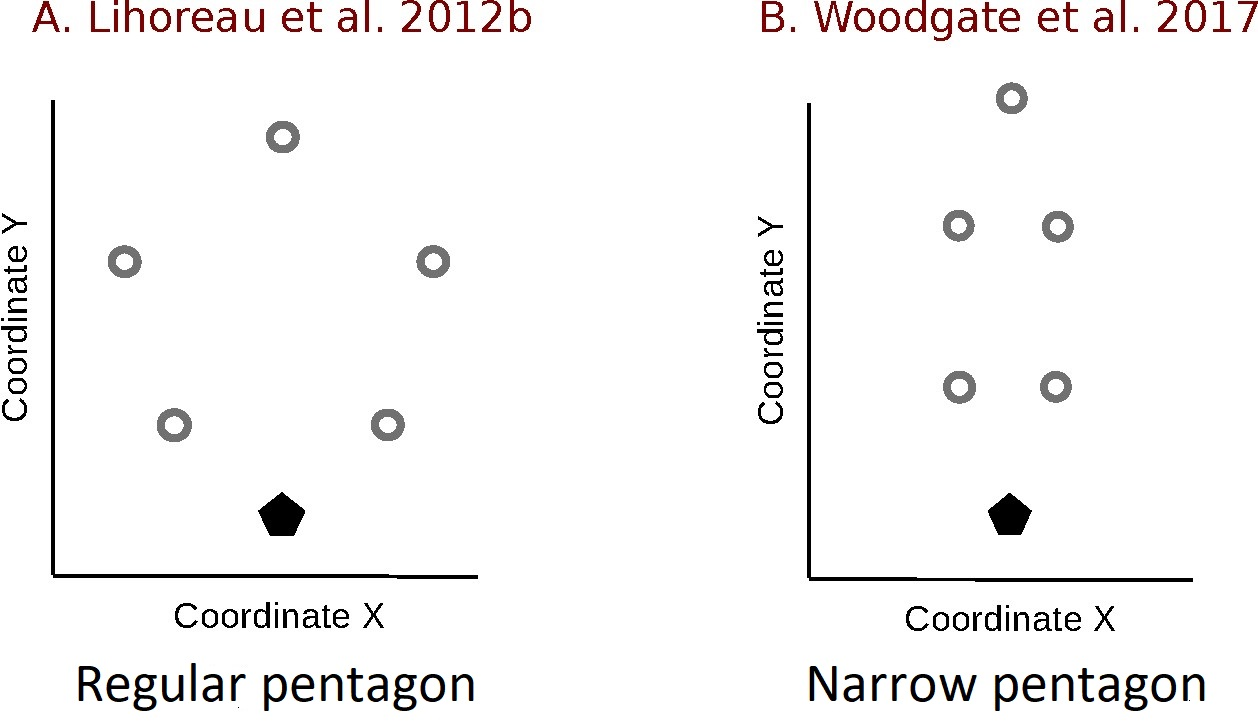

Supplement: S1 Fig — Arrays of artificial flowers (grey circles) and the colony nest (black pentagons) used to obtained the experimental datasets. A. Regular pentagon, modified from Lihoreau et al. [22]. B. Narrow pentagon, modified from Woodgate et al. [13]. (TIF) [file pcbi.1009260.s001.tif]

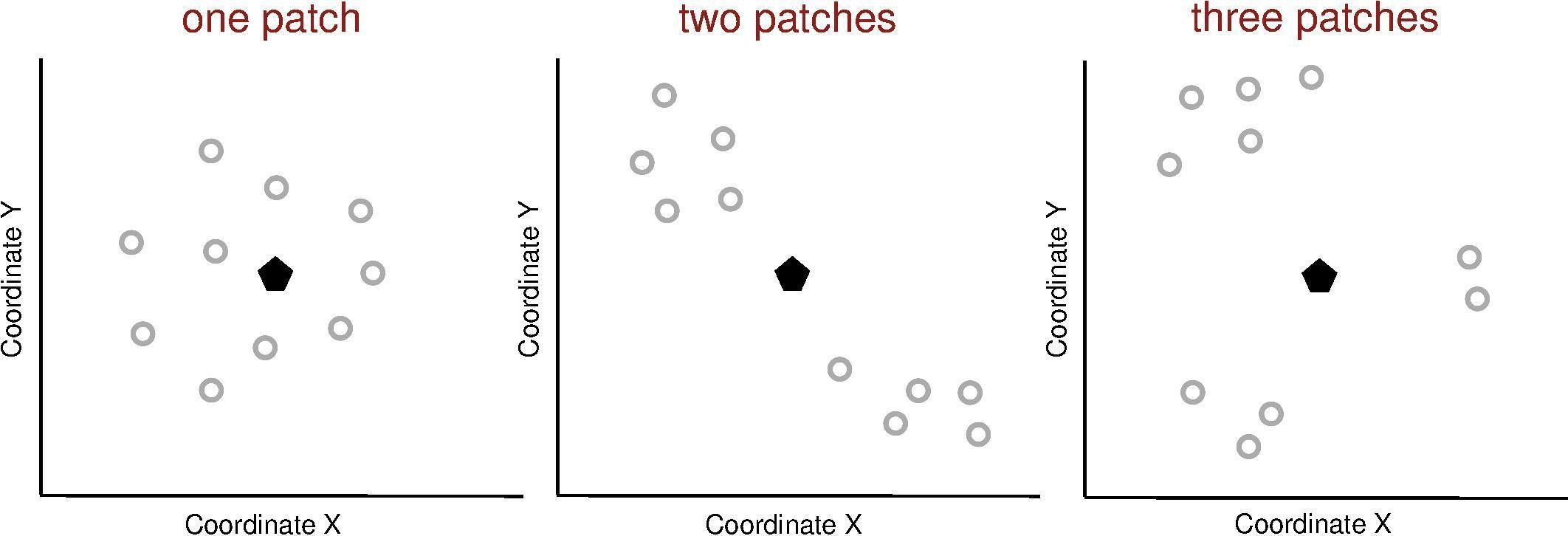

Supplement: S2 Fig — Examples of simulated environments. Spatial distribution of 10 flowers (grey circles) and a colony nest (black pentagon) in three types of environments defined by different levels of flower patchiness. A flower patch was characterized by: 1) a uniform distribution of flowers, 2) a lower distance between flowers within the patch than between all flowers from different patches (see details in methods). (TIF) [file pcbi.1009260.s002.tif]
