# Supplementary material for: A model of resource partitioning between foraging bees based on learning
Source: PLoS Comput Biol. 2021 Jul 28;17(7):e1009260. doi: 10.1371/journal.pcbi.1009260 (PMC8351995; doi:10.1371/journal.pcbi.1009260)
Supplement: S1 Text — (DOCX) [file pcbi.1009260.s003.docx]

**S1 Text. ODD Protocol.**

Below we provide a description of our models following the ODD (Overview, Design concepts, Details) protocol [1,2].

**1 – Purpose and patterns**

The purpose of the models is to offer an explanation to how multiple bees learn to optimize their foraging efficiency. Bees are expected to do so by developing efficient routes (traplines) minimizing spatial overlaps with other foragers (resource partitioning) [3]. We suggest such process can be achieved through combinations of positive and negative reinforcements during flower visits.

The patterns we look at are the development, partial or complete, of stable partitioned routes between flowers by bees, which are observed in most studies on bee foraging strategies. Sensitivity of this behaviour to the parameters of the model is key to determine the usefulness of the model.

**2 - Entities, state variables and scales**

The models depict three kinds of entities: bees, flowers and the colony nest. Bees go out foraging for nectar rewards on flowers and return to the nest. Numbers of bees and flowers can be adjusted in the model. In our simulations we set a ratio of five flowers per bee as to fit the experimental data we replicated [4,5]. A single colony nest is represented, from which all bees forage and come back to. The bees are defined by the following set of state variables:

| Variable name | Variable type and units | Meaning |
| --- | --- | --- |
| *ID* | Integer, constant | A simple number to identify each bee in the model. |
| *Crop* | Integer, dynamic | A count of how many flower rewards (volume of nectar) the bee has collected since its departure from the nest. |
| *maxCrop* | Integer, constant | A maximum number of rewards (volume of nectar) a bee can hold at a time. |
| *probabilityMatrix* | Matrix of probabilities, dynamic | A square matrix of length equal to the sum of number of flowers and the nest, depicting the probability to move between each entity. |
| *indPos* | Integer, dynamic | An integer showing the ID of the flower currently visited by the bee. |
| *winProbabilities* | Float, constant | Value indicative of the probability of winning an encounter on a flower (competition by interference). |
| *indFlowerOutcome* | Matrix of integers, dynamic | A square matrix of length equal to the sum of number of flowers and the nest, depicting a transition’s outcome (1: rewarding; 0: not used; -1: non-rewarding) |

The flowers are static entities placed in the environment. They are defined by:

| Variable name | Variable type and units | Meaning |
| --- | --- | --- |
| *ID* | Integer, constant | A simple number to identify each flower in the model. |
| *resourceOnFlower* | Integer, dynamic | Variable showing the resource availability on the flower (0 or 1). |
| *x,y* | Floats, constant | Coordinates of the flower in the environment. |

The nest is a unique entity which is represented by the following variables:

| Variable name | Variable type and units | Meaning |
| --- | --- | --- |
| *x,y* | Floats, constant | Coordinates of the nest in the environment. |

Both the spatial and temporal scales are represented. Space is represented by the relative distance between the different flowers. Internal parameters define min/max ranges in which each entity can be placed, relative to other entities (see *CreateEnvironment* submodel). Time is represented abstractly. At each step, the bees visit a flower, and possibly feed on it. We assume all travel and flower manipulation times are identical. The use of a movement probability matrix is justified by its capacity to approximate accurately the probability to reach a flower using a random walk, although significantly dependent on what exponent is used in the formula transforming distances in probabilities (See S5 Text for details).

**3 - Process overview and scheduling**

The model covers the execution of a series of foraging bouts (foraging trip starting and ending at the colony nest) by all the bees. During a foraging bout, the model keeps running as long as one bee is foraging. The number of foraging bouts performed by the bees is set as a parameter of the simulation. Each foraging bee chooses its next destination, using the *ChooseDestination* submodel, and updates its position to this new destination. This action is executed in the order of the bees’ *ID* value. If a bee chooses the nest as next destination, its bout is over. Before feeding, the bees resolve any competition occurrences (if more than one bee is on a single flower), in the order of the flower’s *ID* on which the competition occurs. A weighed sampling of the *winProbabilities* decides which individual wins the interaction (default values are uniform for all bees). Bees that either were alone on a flower or won an interaction feed on the flowers, using the *Feeding* submodel. All foraging bees update their movement probability matrix according to their recent experience (details in the *Learning* submodel). Finally, each foraging bee checks if it has reached one of the conditions to return to the nest (crop full or maximum distance of travel), and if so, finishes its foraging bout.

**4 - Design concepts**

*a - Basic principles*

Animals are expected to distribute themselves among different resources to optimize the energetic intake, following what is commonly called the Ideal Free Distribution theory [6]. The environment in which bees forage present multiple constraints. Flowers provide a renewable resource in very low amounts and have a fast turnover changing the distribution of resources. Competition with other bees is strong but also very dynamic as new foragers arrive daily while older ones die.

Bees tend to optimize their foraging activity [3,5,7] by traplining (developing stable routes between feeding sites) and partitioning resources (exploiting feeding sites in different areas of the environment to minimize competition). The aim of the model is to explore the conditions under which traplining emerges from the behaviour of independent bees interacting with the resources and other agents according to the principles of the Ideal Free Distribution. The models reuse parts of a previously published model [5,8] which has been to our knowledge the only model trying to represent the ontogeny of the traplining behaviour to this date.

*b - Emergence*

We focused on how both optimization strategies (traplining and partitioning) can emerge from simple rules of positive and negative reinforcements derived from the foraging experience of individual bees. We explored how these outcomes change when we alter the spatial distribution of the flowers, or remove either type of reinforcement. When bees partition resources, the partitioning index $Q_{norm}$ [9] reaches a maximum value of 1. When bees follow traplines, the cumulated foraging efficiency and route similarity index of the bees approaches a maximum value of 1.

Resource partitioning and traplining are emergent and vary depending on the parameters of the simulation. While traplining could be achieved with a positive reinforcement only, resource partitioning required both positive and negative reinforcements to emerge.

*c - Adaptation*

Bees follow a heuristic in the form of a matrix of movement probability to choose their next destination. This matrix changes with experience to favour the flowers where the bees had a positive experience (collection of nectar). Bees thus learn to fill their nectar crop to capacity. More information on the probability matrix is given in the *ChooseDestination* submodel.

*d - Objectives*

The bees have a unique goal: finding a set amount of nectar to fill their crop (whose capacity is set by the *maxCrop* parameter). In all the conditions explored, this crop capacity was set to 5 resources (units of nectar volume): an arbitrary value used to fit the experimental data of Lihoreau et al. [5]. The decision-making process is altered by this crop filling (described by the *crop* variable) if it reaches the same value as *maxCrop*. If so, the foraging bout is over, and the bee returns to the nest. This behaviour has been observed in experimental conditions [4,5].

*e - Learning*

The learning process occurs as changes made to the probability matrix during the simulation through two components: a positive and a negative reinforcement.

Positive reinforcement is triggered when a bee finds a resource on a flower. When it happens, the bee increases the probability of reusing the transition it just used. Values of the positive reinforcement factor are typically set to be superior or equal to 1. Negative reinforcement is similar but reduces the probability of reuse of the transition. Negative reinforcement occurs when there is either no resource on the flower or if the bee has been evicted from the flower by a competitor. Values of the negative reinforcement factor are typically set to be less than or equal to 1.

*f - Prediction*

In the models the movement probability matrix acts as a prediction tool for the bees. After some experience in the environment, these probabilities become proxies for the probability of finding nectar in flowers, as the positive and negative experiences shape the matrix. The choice of visiting a flower is done prior to the knowledge of presence of a reward on the flower, and only relies on the previous experience on this flower. More information on the movement probability matrix is given in the *ChooseDestination* submodel.

*g - Sensing*

When the model is initialized, the movement probability matrix is based on the distances the bees have to travel between each flower. However, the model does not depict a probability of “not finding any flower”. In experimental situations, it is not rare that the bees would not find all the flowers during their first bout [4,5]. However, in our models it is assumed that the bee always finds a flower, even if it has never visited it. Moreover, it is assumed that the bee knows how to link all the flowers together (knowledge of all the existing transitions) even if these links have never been performed. The use of distance-weighed probabilities is a good approximation of the probabilities obtained by a random walk. As bees always keep tracks of their successive movements after leaving the nest, they can sum these movements to know the direction and approximate distance of the nest.

*h - Interactions*

Two types of interactions were included: exploitation competition and interference competition. Exploitation competition occurs when a bee visits a flower that has already been depleted by a competitor or by itself during a prior visit. Interference competition occurs when two or more bees are simultaneously present on the same flower. When this direct interaction happens, only one bee can access the nectar reward. The winning bee is selected using uniform probabilities.

*i - Stochasticity*

Stochasticity is included in three parts of the model: to place all the flowers in the environment, to choose a bee’s next destination, and to choose a winner from interference competition. When generating a new environment, the submodel *CreateEnvironment* sets rules for placing the flowers. The algorithm will repeatedly try to place a flower at a random position until it fits all the conditions.

The choice of the bee’s next destination (described in the *ChooseDestination* submodel) is done by consulting the movement probability matrix of the agent. As this choice relies on probabilities, we used a stochastic process to choose the next destination, according to the weights of the different destinations’ probabilities. The reason behind this choice is explained in the *Learning* and *Sensing* part of this ODD. Finally, the winner of an interference interaction is decided by choosing with uniform probabilities which bee wins.

*j - Collectives*

The model includes no collectives.

*k - Observation*

There are two observations collected from the bees: visitation sequences and occurrences of competition.

Every time a bee visits a flower during a foraging bout, the flower’s *ID* is reported into a vector containing all the visits in order during the bout. Every time a bee finds an empty flower or finds a competitor on the same flower, these occurrences are reported throughout the foraging bouts. This information gives the number of competitive interactions throughout the bouts. We also save the spatial positions of the flowers to compute the distances between them and find the theoretically optimal routes for the agents. This allows to compute the route qualities of the agents’ successive bouts.

**5 - Initialization**

The first part of the initialization of the models is to create the environment. This part, described in details in the *CreateEnvironment* submodel, places all the flowers and the nest. The environment can be initialized in two ways: either by calling for an existing one (all the environments are stocked in a folder called *Arrays*, inside the main folder where the R script is), or by using the models to generate one.

The variables of the bees that will be dynamic during the simulation are then initialized: *crop*, the number of resources gathered during the ongoing foraging bout; *distanceTravelled* the distance travelled by the bee during this bout; and then *boutFinished*, a Boolean indicating if the bee has finished its bout or not. The movement probability matrix is then initialized for each bee. Finally, we initialize the output objects to store the visitation sequences and resources gathered by each bee during each bout, and store all the information we inputted as parameters to be able to identify what were the parameters used for this simulation later.

**6 - Input data**

The model has no input data.

**7 - Submodels**

Our models can be decomposed in multiple different submodels, which are described here.

*Input Parameters*

The model contains an R script dedicated to the input parameters. While all parameters have comments to help the user, a description of the important parameters is given here.

*environmentType*: Can either contain “generate” or the name of an existing and valid environment found in the *Arrays* folder (the *Arrays* folder will only be created after a first simulation, unless it is created manually beforehand). If using an existing environment, the parameters in part 3.1. will be ignored.

*numberOfResources*: Total number of flowers in the environment.

*numberOfPatches*: In how many patches these flowers should be distributed.

*patchinessIndex*: Deprecated index, should be kept at 1. If multiple patches are created, a value of 1 will ensure the patches are set very distinctly from each other. Values closer to 0 will create a less defined limit between patches. Inputs between 0 and 1 are accepted.

*envSize*: The size of the environment. It defines a square environment in which the flowers will be placed. However, if it is impossible to place all the flowers, this value will be incrementally increased to give enough space.

*flowerPerPatch*: Should contain as many values as the number of patches. If only one patch, it must be set to NULL. Otherwise, it must take a succession of values following the syntax *c(a, b, c, …)*, *a*, *b* and *c* being integer values whose sum equals to *numberOfResources*.

*numberOfArrays*: The number of different environments the model should generate using these parameters.

*reuseGeneratedArrays*: Can take TRUE or FALSE. If TRUE, the model will look into the *Arrays* folder for environments fitting all the parameters. If it does find similar ones, it will use them instead of generating new ones.

*numberOfBees*: Number of bee agents in the model.

*numberOfSimulations*: How many simulations the model will do for each set of parameters.

*numberOfBouts*: How many foraging bouts each bee will do during a simulation.

*distFactor*: Weight given to distance when generating the movement probability matrix. The probability for a transition movement of distance *d* is computed as probability = 1/*d*^*distFactor*. Changing this number will change the initial probability matrix.

*param.useRouteCompare*: deprecated, used to switch between our *Learning* submodel and the route comparison model of [5] and [8]. Should be left at FALSE.

*param.learningFactor*: the value used for the positive reinforcement process. Values should be greater than or equal to 1. Requires at least one value. If multiple values are inputted, the model will run the simulations for each value.

*param.abandonFactor*: The value used for the negative reinforcement process. Values should be between 0 and 1.

*maximumBoutDistance*: Maximum distance a bee can travel during a foraging bout.

In the “Advanced parameters” category different rules can be enforced on the bees. In the following we detail the ones used in our simulations:

*allowNestReturn*: Allows the bee to select the nest as its next destination in the *ChooseDestination* submodel, based on the distance-weighed probabilities. If the bee does so, the foraging bout is finished.

*forbidReverseVector*: This rule forbids the bees to use the reverse movement transition from the one they just used. If the bee has just moved from flower 2 to flower 3, for its next movement this bee will not be given the choice to go from flower 3 to flower 2. This interdiction only applies for the last transition executed.

*onlineReinforcement*: This rule forces the trigger of the *Learning* submodel after each encounter of a flower, instead of only triggering it when the bee had finished its bout. Movement probabilities are thus altered directly after the execution of a movement transition.

*CreateEnvironment*

The creation of an environment happens first in the initialization of the model. The code relating to this process is found in the Functions script, in a function of the same name.

If the user chooses an *environmentType* different than “generate” the model will import the user’s selected environment. The creation of the environment using the “generate” option calls an algorithm we designed to create flower patches. It follows arbitrary rules without any ground in experimental data or theoretical background. In this function, all the parameters inputted in the 3.1.1 part of the Parameters script are being used. Refer to their description in the Input Parameters submodel for their meaning. The basic distance unit between entities is set by an internal parameter, *perceptionRange*, whose default value is 10.

The nest is set first at the centre of the environment (coordinates (0,0)). The different patch centres are placed between 2**perceptionRange* and *envSize* from the nest. Every time a patch is placed, the algorithm checks if this patch centre is at least 16**perceptionRange* away from any other patch centre. The algorithm reiterates this process until the condition is verified. The patch centres act as the first flower of each patch.

Flowers are then placed around the patch centres, respecting the distribution specified in *flowerPerPatch*. All flowers are tentatively placed between 2**perceptionRange* and 4**perceptionRange* of the patch centre, and must verify the condition that each flower has to be at least 2**perceptionRange* from any other flower. The algorithm reiterates this process until the condition is verified. If the algorithm fails to place a flower 200 times in a row, the range at which the flowers can be placed around the patch centre becomes between 2**perceptionRange* and 4**perceptionRange* + (*envSize*/20).

Once all the flowers are placed in all the patches, a table containing the flowers’ *ID*, coordinates *x* and *y*, and the patch they belong to (numbered from 1 to *numberOfPatches*) is created. The nest is also represented in this table, and takes the *ID* 0, and is part of its own patch.

*ChooseDestination*

In order to choose among the possible destinations, the bees refer to a movement probability matrix they are given at the beginning of the simulation. This matrix has *n* rows and columns, *n* being the number of entities (flowers and nest). It is created by extracting in a similar matrix the distance between each entity, and from then applying the following formula for each cell:

(1) $P\left( i\to j \right)=\frac{\frac{1}{{d^{n}}_{ij}}}{\sum_{j}\frac{1}{d_{ij}^{n}}}$

Where $d_{ij}$ is the distance between locations *i* and *j*, and *n* is an integer parameter, whose default value is 2. See the Input Parameters submodel for more information about *distFactor*, which sets the value of *n* in this equation. The probability to go from a flower to itself (immediate revisit) was set to 0. Visiting the same flower twice in a row happens when bees come back to the last departed flower if their search for another flower was unsuccessful, or if they do short orientation flights on the flower. However, these revisits have little importance for the establishment of a stable route [10], and were thus ignored. All rows of the probability matrix are normalised so that their sum is equal to 1. To choose a bee’s next destination, it looks at the matrix’s row matching the flower ID of its current position. As the use of a “reverse transition” is forbidden (see the *forbidReverseVector* parameter described in the Input Parameters submodel), the bee’s previous position is removed from the possible destinations. This prevents an artificial situation the model could create when two flowers are very close to each other, and the probability to move between them is much higher than any other probability. Without this rule, bees would often get stuck navigating back and forth between both flowers. If the *allowNestReturn* is used, the nest is kept in the possible destinations. Otherwise, it is removed. A weighed sample is made between all the remaining potential destinations to choose the one that the bee will use.

*Feeding*

This submodel takes care of all matters that happen when a bee lands on a flower, i.e. all competition occurrences and the collection of resources. If two or more bees choose the same destination on the same step, one of them is chosen randomly to access to the resource. The losing bees will still depart from this flower for the next step, but will not feed.

*Learning*

As bees finish to go through the *Feeding* module, they have five possible outcomes: (i) the bee has landed alone on a flower, and found resources; (ii) the bee has landed alone on a flower, and did not find any resource; (iii) the bee has landed with competitors on a flower, and has lost the competition; (iv) the bee has landed with competitors on a flower, has won the competition, but found no resource; (v) the bee has landed with competitors on a flower, has won the competition, and found resources. These outcomes can be placed into two categories: the positive (i and v) or negative (ii, iii and iv) outcomes.

Each bee has a square matrix named *indFlowerOutcome*, with *n* rows and columns, *n* being the combined number of flowers and nest. Every bout it is initialized with 0s in all cells, and then altered every time a transition is used during the bout. If the outcome of the transition performed by the bee is positive (rewarding), the cell corresponding to this transition in the *indFlowerOutcome* matrix takes a value of 1. Similarly, if the outcome in negative (non-rewarding), it takes the value -1. Only the first use of a transition during a bout will alter this matrix. This matrix is then used as a reference to change the movement probability matrix as it contains a trace of all the transitions that receive a change.

**References**

1. Grimm V, Berger U, Bastiansen F, Eliassen S, Ginot V, Giske J, et al. A standard protocol for describing individual-based and agent-based models. Ecol Modell. 2006;198(1–2): 115–126. doi: 10.1016/j.ecolmodel.2006.04.023

2. Grimm V, Railsback SF, Vincenot CE, Berger U, Gallagher C, Deangelis DL, et al. The ODD protocol for describing agent-based and other simulation models: A second update to improve clarity, replication, and structural realism. Jasss. 2020;23(2): 7. doi: [10.18564/jasss.4259](http://doi.org/10.18564/jasss.4259)

3. Lihoreau M, Chittka L, Raine NE. Monitoring Flower Visitation Networks and Interactions between Pairs of Bumble Bees in a Large Outdoor Flight Cage. PLoS One. 2016;11(3): e0150844. doi: [10.1371/journal.pone.0150844](https://doi.org/10.1371/journal.pone.0150844)

4. Woodgate JL, Makinson JC, Lim KS, Reynolds AM, Chittka L. Continuous Radar Tracking Illustrates the Development of Multi-Destination Routes of Bumblebees. Sci Rep. 2017;7(1): 17323. doi: [10.1038/s41598-017-17553-1](https://doi.org/10.1038/s41598-017-17553-1)

5. Lihoreau M, Raine NE, Reynolds AM, Stelzer RJ, Lim KS, Smith AD, et al. Radar Tracking and Motion-Sensitive Cameras on Flowers Reveal the Development of Pollinator Multi-Destination Routes over Large Spatial Scales. PLoS Biol. 2012;10(9): 19–21. doi: [10.1371/journal.pbio.1001392](https://doi.org/10.1371/journal.pbio.1001392)

6. Fretwell SD. On territorial behavior and other factors influencing habitat distribution in birds. Acta Biotheor. 1969;19(1): 16–36. doi: 10.1007/BF01601955

7. Ohashi K, Thomson JD, D’Souza D. Trapline foraging by bumble bees: IV. Optimization of route geometry in the absence of competition. Behav Ecol. 2007;18(1): 1–11. doi: [10.1093/beheco/arl053](http://doi.org/10.1093/beheco/arl053)

8. Reynolds AM, Lihoreau M, Chittka L. A Simple Iterative Model Accurately Captures Complex Trapline Formation by Bumblebees Across Spatial Scales and Flower Arrangements. PLoS Comput Biol. 2013;9(3): e1002938. doi: [10.1371/journal.pcbi.1002938](https://doi.org/10.1371/journal.pcbi.1002938)

9. Pasquaretta C, Jeanson R. Division of labor as a bipartite network. Behav Ecol. 2018;29(2): 342–352. doi: [10.1093/beheco/arx170](http://doi.org/10.1093/beheco/arx170)

10. Lihoreau M, Chittka L, Raine NE. Travel optimization by foraging bumblebees through readjustments of traplines after discovery of new feeding locations. Am Nat. 2010;176(6): 744–757. doi: [10.1086/657042](https://doi.org/10.1086/657042)
