# Supplementary material for: A model of resource partitioning between foraging bees based on learning
Source: PLoS Comput Biol. 2021 Jul 28;17(7):e1009260. doi: 10.1371/journal.pcbi.1009260 (PMC8351995; doi:10.1371/journal.pcbi.1009260)
Supplement: S2 Text — (DOCX) [file pcbi.1009260.s004.docx]

**S2 Text. Sensitivity analysis of positive and negative reinforcements.**

We ran a sensitivity analysis for the two main parameters: the positive and negative reinforcements. As we had no *a priori* understanding of how the model behaved with different values of reinforcements, we ran simulations on ranges of positive (1, 1.2, 1.4, 1.6, 1.8, 2) and negative (0, 0.1, 0.2, 0.3, 0.4, 0.5, 0.6, 0.7, 0.8, 0.9, 1) reinforcements for a total of 66 sets of parameters, for each environment type (one, two and three patches; S2 Fig). We simulated 10 environments for each environment type and computed 100 simulations of 50 foraging bouts per iteration (i.e. 1000 simulations per environment type and set of parameters).

Since our study focused on resource partitioning, we extracted the $Q_{norm}$ index values for these simulations and compared them. We plotted a heatmap showing the value of the mean $Q_{norm}$ index at the last foraging bout for each set of parameters and environment type (Fig A). In all types of environments, positive reinforcement had a strong effect on the final $Q_{norm}$ index value. High values of resource partitioning were obtained for positive reinforcement values > 1.5. By contrast, negative reinforcement only had an impact in environments with two or three patches. High values of resource partitioning were obtained for negative reinforcement values larger than 0.75.

For each pair of bees, we also looked at how this same index evolved over successive foraging bouts. Fig B shows the dynamics of mean $Q_{norm}$ index across 50 foraging bouts for each combination of positive reinforcement (1.0, 1.2, 1.4, 1.6, 1.8, 2.0) and negative reinforcement (0, 0.1, 0.2, 0.3, 0.4, 0.5, 0.6, 0.7, 0.8, 0.9, 1) parameters, and each environment type (one patch, two patches, three patches). Higher values of both positive and negative reinforcements most often lead to faster resource partitioning (with some uncertainty due to the probabilistic nature of the model). Combinations of values in which the negative reinforcement factor was missing (violet gradient curves) led to a decrease in partitioning.

Finally, we also looked at how the Similarity Index was affected by these ranges of parameters. We drew a similar heatmap showing the Similarity Index at the last foraging bout for each parameter set and environment type (Fig C). It appeared that positive reinforcement had a strong impact on route similarity in all environment types, while negative reinforcement only seemed to have a small effect only on the two and three patches environments.

**
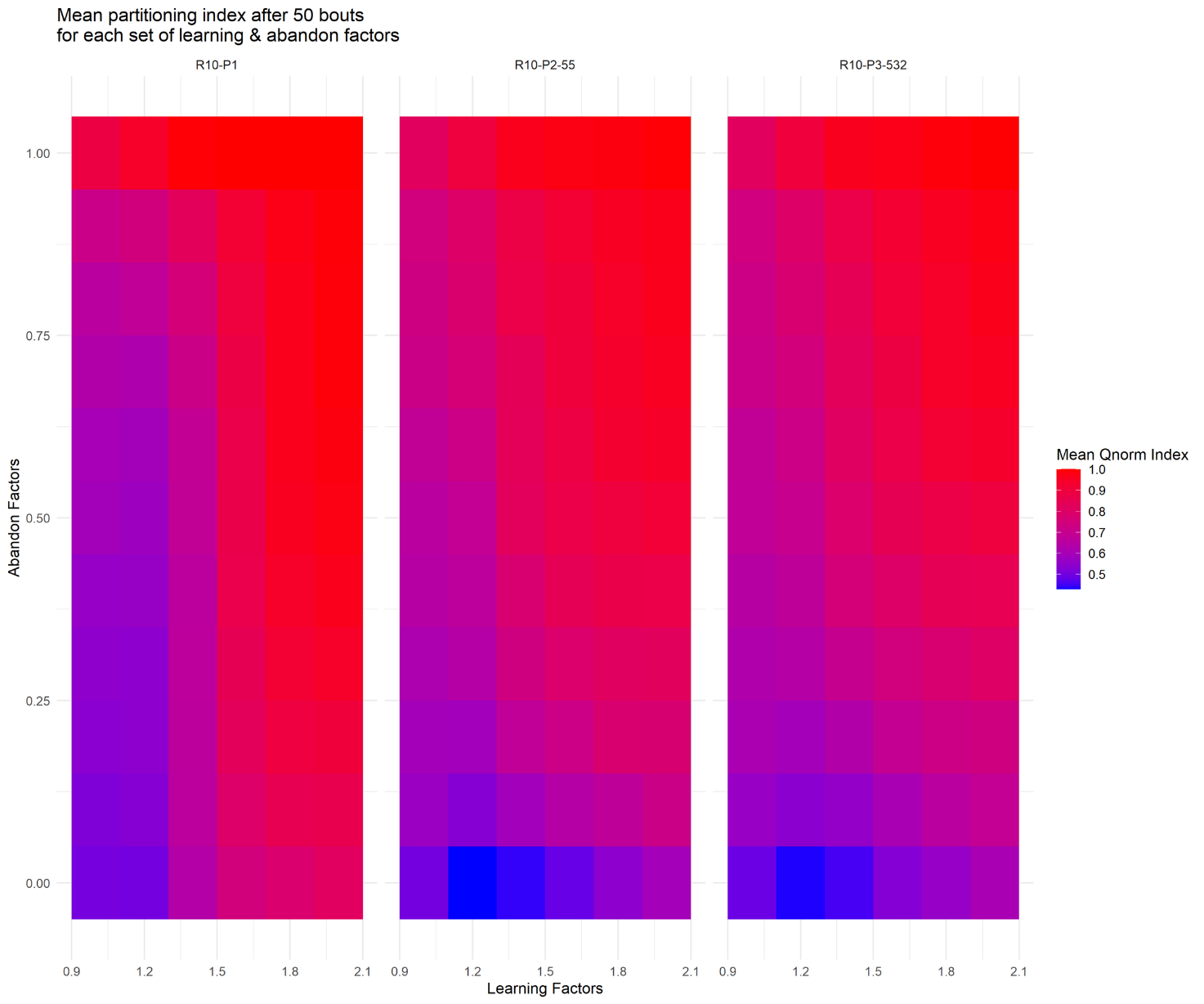
**

**Fig A.** **Heatmap graph of sensitivity analysis of partitioning index to parameters.** Heatmap showing the mean $Q_{norm}$ Index value after 50 foraging bouts (mean over 1000 simulations on 10 arrays of the same environment type), for each combination of positive reinforcement (1.0, 1.2, 1.4, 1.6, 1.8, 2.0) and negative reinforcement (0, 0.1, 0.2, 0.3, 0.4, 0.5, 0.6, 0.7, 0.8, 0.9, 1) parameters, and for each environment type (one patch, two patches, three patches). For simplicity, we inverted the values of negative reinforcement. 0 indicate models without negative reinforcement.


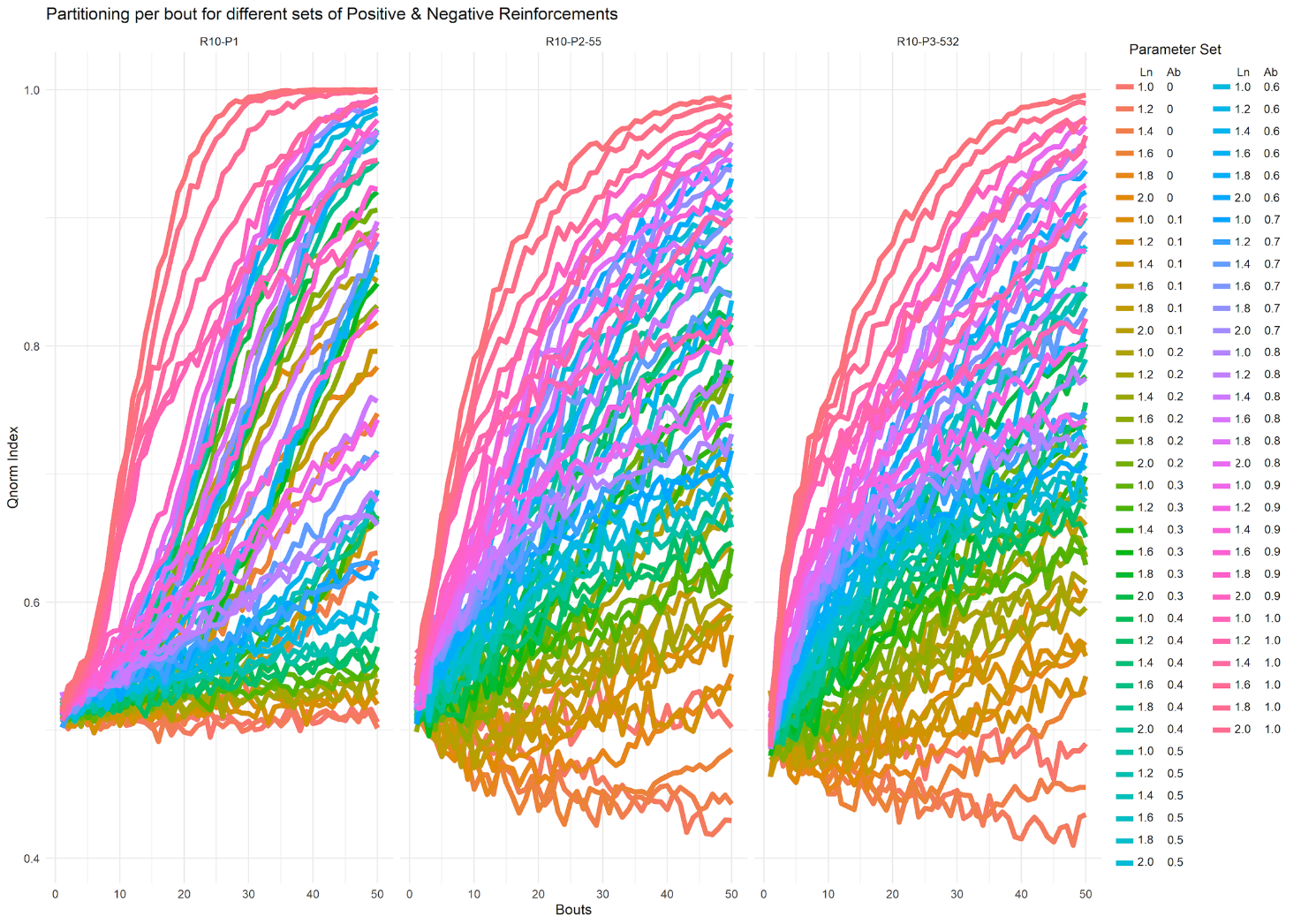


**Fig B.** Dynamic of the mean $Q_{norm}$ Index across foraging bouts for each combination of positive (1.0, 1.2, 1.4, 1.6, 1.8, 2.0) and negative (0, 0.1, 0.2, 0.3, 0.4, 0.5, 0.6, 0.7, 0.8, 0.9, 1) reinforcement factors and for each environment type (one patch, two patches, three patches). For simplicity, we inverted the values of negative reinforcement here. 0 being models without negative reinforcement.


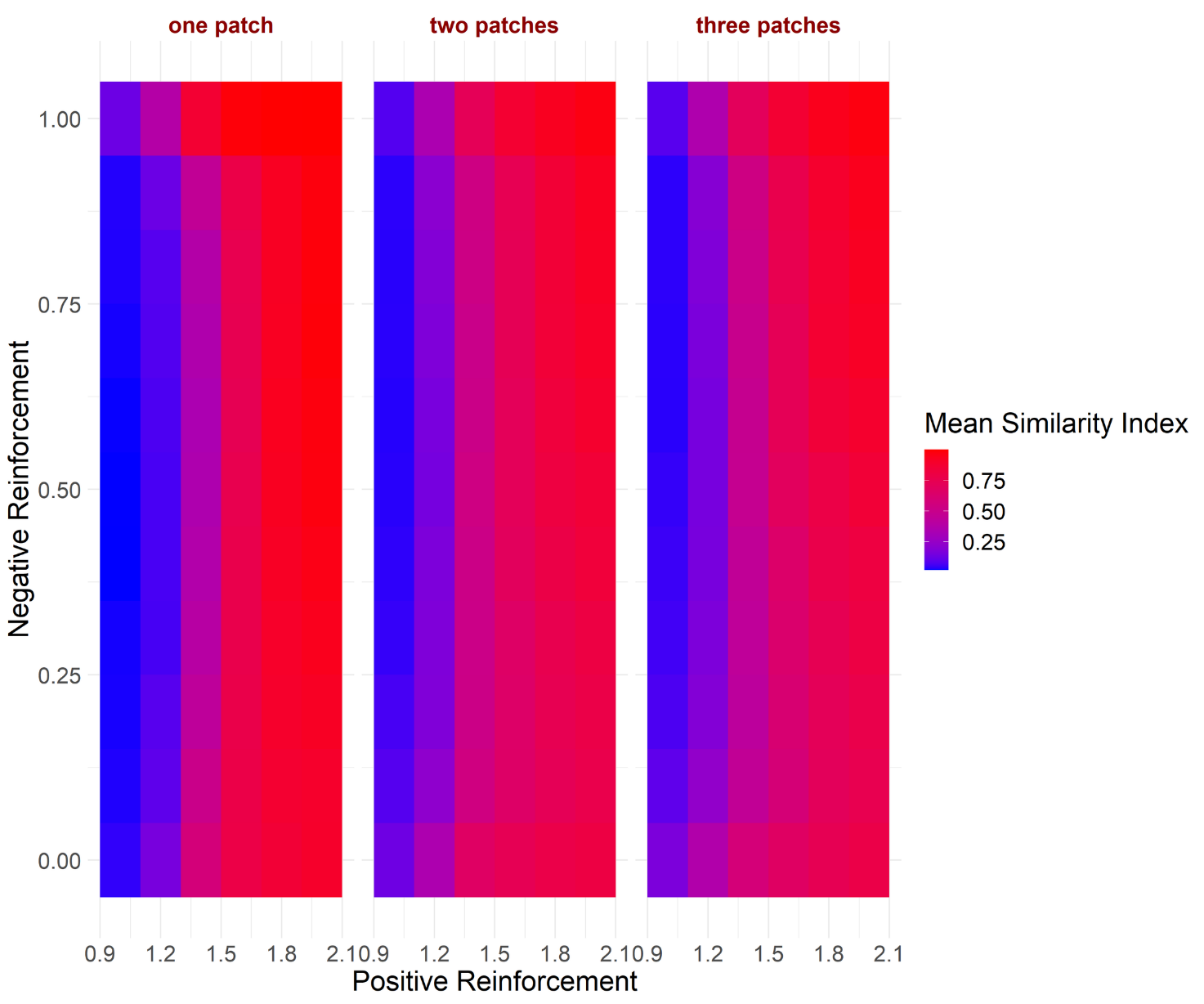


**Fig C.** Heatmap showing the mean Similarity Index value after 50 foraging bouts (mean over 1000 simulations on 10 arrays of the same environment type), for each combination of positive reinforcement (1.0, 1.2, 1.4, 1.6, 1.8, 2.0) and negative reinforcement (0, 0.1, 0.2, 0.3, 0.4, 0.5, 0.6, 0.7, 0.8, 0.9, 1) parameters, and for each environment type (one patch, two patches, three patches). For simplicity, we inverted the values of negative reinforcement. 0 indicate models without negative reinforcement.
