# Supplementary material for: A model of resource partitioning between foraging bees based on learning
Source: PLoS Comput Biol. 2021 Jul 28;17(7):e1009260. doi: 10.1371/journal.pcbi.1009260 (PMC8351995; doi:10.1371/journal.pcbi.1009260)
Supplement: S3 Text — (DOCX) [file pcbi.1009260.s005.docx]

**S3 Text. Qualitative comparison between simulations and observations in the narrow pentagon.**

We ran simulations with one forager to compare model outcomes to observational data using a second reference field study [1]. In this study, the authors used five artificial flowers arranged in a narrow pentagon (S1B Fig). Four bumblebees were tested during 27 to 61 foraging bouts each (flower visitation sequences were kindly provided by Joe Woodgate). One of these bumblebees was tested over different days and was therefore removed from the analyses (as bees’ experience memory drops overnight [2]). In these conditions, none of the bumblebees developed a stable trapline, although all significantly increased their foraging efficiency with time (e.g. reduced travel distance and duration, increased similarity between two consecutive flower visitation sequences).

The sole implementation of positive reinforcement in Model 1[+] was sufficient to replicate the observations. While the use of the negative reinforcement alone in Model 2[-] showed drastically different results, its addition with the positive reinforcement in Model 3[+\-] had no major effect on route quality nor on route similarity trends (Fig A). Overall, simulations of Models 1[+] and 3[+\-] showed good qualitative fit to the traplining behaviour observed in real bees – i.e. there is a trend of increasing route similarities across foraging bouts. Note however that the models tend to overestimate the bee ability to develop stable routes. This imperfect match could be due to the low amount of available experimental data in the original study (three individuals in [1]). Alternately, the model has been shown to overestimate the increase of the similarity index and underestimate the initial similarity of the first few bouts (See Discussion).


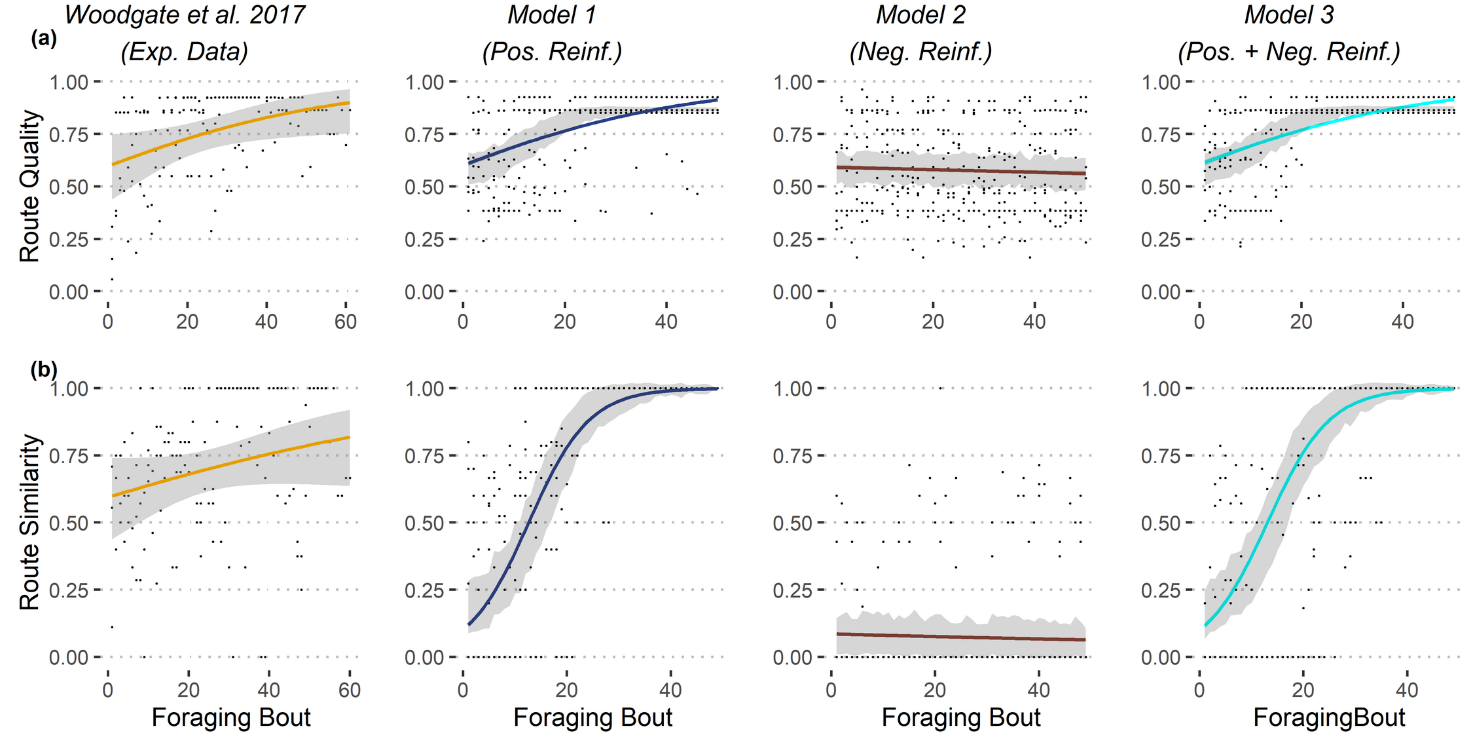


**Fig A.** Qualitative comparisons of route qualities (A) and similarities (B) between simulations and experimental data (narrow pentagon array of flowers as in [1]) for one forager (see details of the models in Fig 1). For each dataset, we show the estimated average trends across foraging bouts (colored curves), along with the standard error (gray areas). For the sake of eye comparison, in the simulation plots the standard error of the mean is computed from a sample of 3 simulations (N = 3 bees in our sample taken from [1]). Average trends were estimated over 500 simulation runs, using GLMM Binomial model with bee identity as random effect (bee identity nested in simulation identity for simulated data).

**References**

1. Woodgate JL, Makinson JC, Lim KS, Reynolds AM, Chittka L. Continuous Radar Tracking Illustrates the Development of Multi-Destination Routes of Bumblebees. Sci Rep. 2017;7(1): 17323. doi: [10.1038/s41598-017-17553-1](https://doi.org/10.1038/s41598-017-17553-1)

2. Lihoreau M, Chittka L, Raine NE. Travel optimization by foraging bumblebees through readjustments of traplines after discovery of new feeding locations. Am Nat. 2010;176(6): 744–757. doi: [10.1086/657042](https://doi.org/10.1086/657042)
