# Supplementary material for: A model of resource partitioning between foraging bees based on learning
Source: PLoS Comput Biol. 2021 Jul 28;17(7):e1009260. doi: 10.1371/journal.pcbi.1009260 (PMC8351995; doi:10.1371/journal.pcbi.1009260)
Supplement: S4 Text — (DOCX) [file pcbi.1009260.s006.docx]

**S4 Text. Predictions with more than two bees.**

We explored the emergence of resource partitioning in groups of 5 bees, and how this varies in environments containing 20, 25, 30, 40, 50, 70 and 100 flowers, thus encompassing a gradient of competition pressures from conditions where there are not enough flowers for all bees (20) to conditions where there are four times more flowers than necessary for all bees (100). For simplicity, flowers were evenly distributed (i.e. environment with one patch). The model used for these simulations is model 3[+/-]. For each flower density, we generated 10 environments, and ran 100 simulations of 100 foraging bouts, for a total of 1000 simulations per density value. We computed the resource partitioning index ($Q_{norm}$) at each foraging bout.

The mean final $Q_{norm}$ was higher in environments with most flowers (Fig A). Plotting the mean final partitioning index (final foraging bout) as a function of the number of available flowers confirmed that bees converge to a plateau when increasing the number of flowers up until around 50 flowers (Fig A). As the number of flowers increases, positive reinforcement became more prevalent in driving partitioning, while negative reinforcement became less relevant. This result simply reflects how unlikely it becomes to come across competition as the resources become more available.


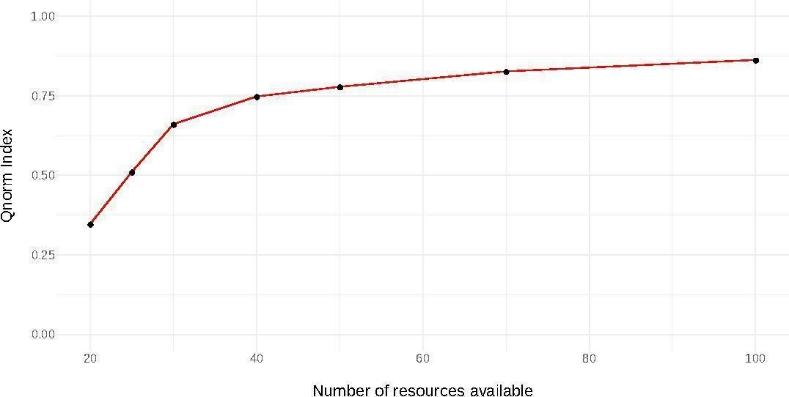


**Fig A.** Evaluation of the mean final $Q_{norm}$ index (after 100 foraging bouts) as a function of increase resources availability. The model run has the positive reinforcement factor set at 1.5, and the negative reinforcement factor set at 0.75 (model 3[+\-]) with five bees foraging in environments of one patch.
