# Supplementary material for: A model of resource partitioning between foraging bees based on learning
Source: PLoS Comput Biol. 2021 Jul 28;17(7):e1009260. doi: 10.1371/journal.pcbi.1009260 (PMC8351995; doi:10.1371/journal.pcbi.1009260)
Supplement: S5 Text — (DOCX) [file pcbi.1009260.s007.docx]

**S5 Text. Supplementary information on the similarity index.**

We used a route similarity index between two consecutive visitation sequences in order to assess how similar they were. We designed an index that would account for both the similarity of transitions used, but also the number of transitions used. For the computation of this index, visitation sequences are decomposed into smaller sequences, whose length is set by a parameter of the function. In our study, we set this value arbitrarily to 3. These smaller sequences are made by sliding a window of the specified length through the visitation sequence, moving the window 1 visit further each time (see example below).

The compared sequences, *a* and *b*, are thus decomposed into small sequences of the defined length after excluding the nest position. The number of similar small sequences used in both sequences are stored in an object called $s_{ab}$. All uses of these common small sequences in the sequences *a* and *b* are then highlighted, and all flower visits highlighted as such are counted, and stored in an object called $s_{ab}$. The longest sequence between *a* and *b* has its number of visits stored in a second object called $l_{ab}$. The similarity index is then calculated using the formula:

$${SI}_{ab}=\frac{s_{ab}}{{2l}_{ab}}$$

Which represents the number of visits part of common small sequences ($s_{ab})$ divided by the total number of visits in both sequences (${2l}_{ab}$). This multiplication by 2 in the denominator allows for accounting in length differences between the two compared sequences.

Example:

We retrieved two visitation sequences *a* and *b* from successive bouts:

Sequence *a*: N 5 3 4 N

Sequence *b*: N 5 3 4 2 5 3 4 N

First, the visits to the nest are removed, giving the following sequences:

Sequence *a*: 5 3 4

Sequence *b*: 5 3 4 2 5 3 4

Then, the small sequences used in both sequences are identified:

| Small sequences | Used in seq. *a* ? | Used in seq. *b* ? |
| --- | --- | --- |
| 5 ➝ 3 ➝ 4 | Yes | Yes |
| 3 ➝ 4 ➝ 2 | No | Yes |
| 4 ➝ 2 ➝ 5 | No | Yes |
| 2 ➝ 5 ➝ 3 | No | Yes |

The two sequences show a common small sequence: 5-3-4. The uses of this triplet in the sequences *a* and *b* is highlighted (here in bold):

Sequence *a*: **5 3 4**

Sequence *b*: **5 3 4** 2 **5 3 4**

In this case, 9 total visits are part of repeated sequences. The longest sequence, *b*, has a length of 7 visits. Thus, the computation of our index is:

$${SI}_{ab}=\frac{s_{ab}}{{2l}_{ab}}= \frac{9}{2*7}=0.643$$
