# Supplementary material for: A model of resource partitioning between foraging bees based on learning
Source: PLoS Comput Biol. 2021 Jul 28;17(7):e1009260. doi: 10.1371/journal.pcbi.1009260 (PMC8351995; doi:10.1371/journal.pcbi.1009260)
Supplement: S6 Text — (DOCX) [file pcbi.1009260.s008.docx]

**S6 Text. Details on the movement probability matrix**

In our model, the agent bees rely on a movement probability matrix to navigate from one flower to the other. This design was kept from the initial model developed in [1]. To obtain such a matrix, the coordinates of all flowers and nest are used to compute the distance between each pair of entities. From there, the probability *P* to go from a flower *i* to a flower *j* is determined by the following formula:

$P\left( i\to j \right)=\frac{\frac{1}{{d^{n}}_{ij}}}{\sum_{j}\frac{1}{d_{ij}^{n}}}$

where $d_{ij}$is the distance between the flower *i* and *j*, and *n* an exponent arbitrarily inserted to change the way distance would affect probabilities. This design was chosen because it approximated closely the probabilities to find the different flowers when using a simple random walk.

To present their similarities, we compared the probabilities obtained by both our probability matrix and a lattice-based random walk. We used the regular pentagon used for [1] as an example for this comparison, with all flower positions rounded to the nearest integer for simplicity. We simulated 10000 bees leaving the nest, with the goal to find any flower in 5000 steps. In each step, an agent had 4 choices, to go up or down on either the *x* or *y* abscisses, with the same probability. The simulation stopped if the bee got at a distance of 5 metres or less than a flower, thus mimicking a perception range. The results of this comparison are presented in Fig A.

The results in Fig A show that as the exponent *n* increases, the probabilities to do the shortest movements (towards flowers 1 and 5) increases while those of longer movements (towards flowers 2, 3 and 4) decreases. In this specific environment, an exponent of 3 would replicate most accurately the initial probabilities of encountering each flower using a random walk. In our study, however, we did set the value of this exponent to 2 throughout all simulations, as it was the value used in the previous paper using this method [1].

**
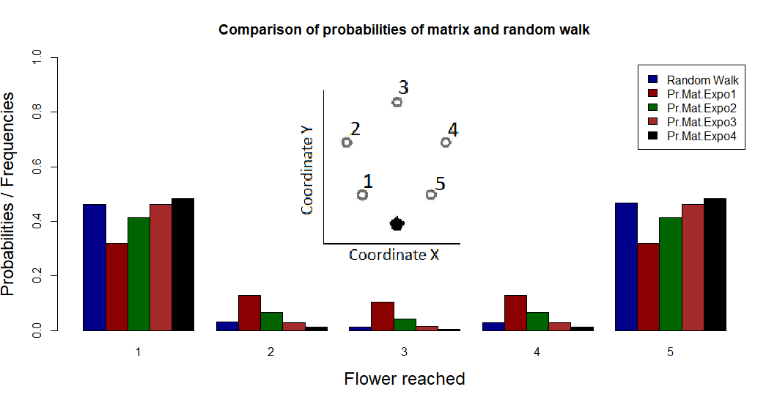
**

**Fig A.** Comparison of probabilities of reaching any flower (numbered circles) from the nest (black pentagon) using either a random walk or the probability matrix used in the models. For the probability matrix, 4 distinct parameter values were tested for the exponent found in the equation used to compute the transition probabilities.

**References**

1. Reynolds AM, Lihoreau M, Chittka L. A Simple Iterative Model Accurately Captures Complex Trapline Formation by Bumblebees Across Spatial Scales and Flower Arrangements. PLoS Comput Biol. 2013;9(3): e1002938. doi: [10.1371/journal.pcbi.1002938](https://doi.org/10.1371/journal.pcbi.1002938)

2. Lihoreau M, Raine NE, Reynolds AM, Stelzer RJ, Lim KS, Smith AD, et al. Radar Tracking and Motion-Sensitive Cameras on Flowers Reveal the Development of Pollinator Multi-Destination Routes over Large Spatial Scales. PLoS Biol. 2012;10(9): 19–21. doi: [10.1371/journal.pbio.1001392](https://doi.org/10.1371/journal.pbio.1001392)
