# Supplementary material for: A model of resource partitioning between foraging bees based on learning
Source: PLoS Comput Biol. 2021 Jul 28;17(7):e1009260. doi: 10.1371/journal.pcbi.1009260 (PMC8351995; doi:10.1371/journal.pcbi.1009260)
Supplement: Attachment — Submitted filename: Response to reviewers.docx [file pcbi.1009260.s010.docx]

Editors’ Letter:

Dear Mr. DUBOIS,

Thank you very much for submitting your manuscript "A model of resource partitioning between foraging bees based on positive and negative associations" for consideration at PLOS Computational Biology.

As with all papers reviewed by the journal, your manuscript was reviewed by members of the editorial board and by several independent reviewers. In light of the reviews (below this email), we would like to invite the resubmission of a significantly-revised version that takes into account the reviewers' comments.

We cannot make any decision about publication until we have seen the revised manuscript and your response to the reviewers' comments. Your revised manuscript is also likely to be sent to reviewers for further evaluation.

When you are ready to resubmit, please upload the following:

[1] A letter containing a detailed list of your responses to the review comments and a description of the changes you have made in the manuscript. Please note while forming your response, if your article is accepted, you may have the opportunity to make the peer review history publicly available. The record will include editor decision letters (with reviews) and your responses to reviewer comments. If eligible, we will contact you to opt in or out.

[2] Two versions of the revised manuscript: one with either highlights or tracked changes denoting where the text has been changed; the other a clean version (uploaded as the manuscript file).

Important additional instructions are given below your reviewer comments.

Please prepare and submit your revised manuscript within 60 days. If you anticipate any delay, please let us know the expected resubmission date by replying to this email. Please note that revised manuscripts received after the 60-day due date may require evaluation and peer review similar to newly submitted manuscripts.

Thank you again for your submission. We hope that our editorial process has been constructive so far, and we welcome your feedback at any time. Please don't hesitate to contact us if you have any questions or comments.

Sincerely,

Phillip Staniczenko
Guest Editor
PLOS Computational Biology

Stefano Allesina
Deputy Editor
PLOS Computational Biology

Dear Editor,

Thank you for inviting us to revise our manuscript. The two reviewers made useful comments, which we believed helped us improve the text. Please find attached an extensively revised version taking addressing all the reviewers’ comments.

We made a particular effort to clarify different aspects of the model and justify our choices (e.g. vector-based learning, distance matrix for initial flower visits). We simplified the presentation of the results by relabelling the models (now model 1[+], model 2[-], model 3[+/-]) and moving the results of the statistical analyses in tables (see new Tables 1 and 2). We also now discuss in more details the influence of positive and negative reinforcements on route similarity in a new paragraph (Lines 295-305) backed up with a new sensitivity analysis (Supplementary Materials, Appendix S2, Lines 331-336, and new Figure S5).

You will find below our answers pasted below each comment.

We hope you will find this revised version acceptable for publication.

With kind regards,

Thibault Dubois

**Reviewer #1**

This study explores how two possible mechanisms – positive reinforcement and negative reinforcement – might cause the development of traplines and resource partitioning in foraging bumblebees. The authors have developed an individual based model in which the probability of movement between any two flowers is given in a matrix for each bee. The bee moves from flower to flower and each time a bee visits a flower, the probability associated with that movement is updated according to the mechanism(s) used. The authors compare three versions of their model: with positive reinforcement only, with negative reinforcement only, and with both types of reinforcement.

The authors find that, for two bees foraging at the same time, these mechanisms have different effects in different environments (number of patches). In two or three patch environments, positive reinforcement alone allows for traplining but not resource partitioning, while negative reinforcement alone allows for resource partitioning but not traplining. When both are combined, traplining and resource partitioning result. In a single patch environment, negative reinforcement alone is not sufficient for either traplining or resource partitioning. Positive reinforcement alone or positive reinforcement and negative reinforcement combined allow for both traplining and resource partitioning.

I find the study to be well grounded in the literature on trapline formation, learning, and navigation in bumblebees. The authors make use of two previously published experimental studies (of trapline formation in bumblebees foraging on artificial arrays of flowers) for model validation. I have however, provided (below in the detailed comments) a few additional bee movement modelling references that the authors may use to support their claims and better situate their work within the modelling literature.

> Thank you for the positive words and the useful suggestions. We explain below how we have dealt with each of your comments.

**General Comments**

I find this to be an interesting and compelling model that adheres to the modelling principles of parsimony specificity. The authors avoid making the model needlessly complex and include only behavioural details that are relevant to the research question and well enough understood to be confidently modelled, but they include enough detail of the relevant behaviours to be able to fully address the research question. To my knowledge, there are no other bee movement models which address resource partitioning, and indeed the number of bee models of any learning behaviour at all is limited, making this an excellent addition to the literature.

I find, however, that the introduction of the paper and the presentation of the results sets up the reader to expect a greater treatment of traplining (route similarity) in the discussion. The combination of the effects of the positive and negative reinforcements in different landscapes with both resource partitioning and route similarity (as I have summarized above) presents a more complex, and thus more interesting, picture of the results of the model. I would suggest that the authors include more to this effect in the discussion section.

> We agree and have added a whole new paragraph in the discussion. We also extended the sensitivity analysis to the route similarity index (see our answer to your comment 1.13). Text lines 295-305 now reads : « We then used our models for predicting behaviours of two competing bees in different types of environments. To develop a trapline in this competitive situation, the bees needed to find rewarding flowers but also avoid competitors. These two goals were independently fulfilled by the positive reinforcement and the negative reinforcement. Simulated bees were fastest to develop a trapline when using the positive reinforcement only, and unable to follow any stable route when solely using the negative reinforcement. However, this does not indicate that the use of both reinforcements was less effective than just positive reinforcement. Simulated bees were indeed more likely to establish a stable route with positive reinforcement only, but these routes most likely contained contested flowers that the bees were not able to give up on, as they did not change their behaviour after experiencing unrewarded visits. This assumption is comforted by the fact that both reinforcements leads to a greater resource partitioning and a higher collective foraging efficiency. »

**Detailed Comments**

**Comment 1.1 :**

Line 94-95. What is vector-based learning ? If I understand correctly it refers to the fact that the bees are remembering the vectors between significant locations, rather than the entire path they have followed in each foraging bout. This should be clarified here.

> This is correct. Text lines 95-97 now reads « To address this issue, it was proposed that trapline formation could also emerge from vector-based learning [24], in which the bee remembers independent vectors instead of complete routes.».

**Comment 1.2 :**

Throughout lines 84-113, I found myself getting confused about route-based learning and vector-based learning. In Lines 101 and 110 you use the term ‘route learning’ in reference to your model, which uses vector-based learning. Perhaps ‘trapline formation’ would be a better term here ?

> We agree and replaced mentions of “route learning” by “trapline formation” throughout.

**Comment 1.3 :**

Line 96-98. I suggest a few bee population/movement modelling papers which could provide broader support for your claims here than just relying on the BEEHAVE suite of models, although you may be able to find others.

H. Qu and F. Drummond. Simulation-based modelling of wild blueberry pollination. Computers and Electronics in Agriculture, 144 :94–101, 201

J. Everaars, J. Settele, and C. F. Dormann. Fragmentation of nest and foraging habitat affects time budgets of solitary bees, their fitness and pollination services, depending on traits : Results from an individual-based model. PLOS One, 13(2) :e0188269, 2018.

S. A. Rands. Landscape fragmentation and pollinator movement within agricultural environments : a modelling framework for exploring foraging and movement ecology. PeerJ, 2 :e269, 2014.

> Thanks for the suggestions. We now refer to these papers in the text (Line 100) and the references (Lines 667-675).

**Comment 1.4 :**

Line 101. Do you mean comparing the models to each other ? Or to data ? Both ?

> The sentence (Lines 102-104) now reads : « Here, we investigated the behavioural mechanisms underlying resource partitioning among traplining bees by comparing predictions of three agent-based models to each other. The different models integrate learning behaviour and social interactions in slightly different ways. ».

**Comment 1.5 :**

Lines 137-139. You explain later in the manuscript that the sample size in the narrow pentagon array study was too small to allow for quantitative comparison, but I feel that that information should be presented here, otherwise the reader is left wondering why only qualitative comparison was possible.

> Text lines 141-144 now reads : « While Woodgate *et al.* [13] tested six bees in a narrow pentagonal array (Fig S1B), only three of them presented enough successive foraging bouts in a single day to allow statistical comparisons with our model. Thus, for this dataset only qualitative comparisons were made with the model simulations. »

**Comment 1.6 :**

Lines 160-169. Here I wondered, if you decrease the positive reinforcement does that make the model match the data better ? Since this is the main validation of the model, I would like to see a greater discussion of the discrepancy between the model and the data. This would easily be done by incorporating the discussion from S3 into the main manuscript.

> Decreasing the positive reinforcement did not improve the match for the route similarity data (the fit of the route quality was even worse). While the curve of the route similarity changes with lower reinforcement, its origin is always lower in the model. When exploring their environment, especially as naive foragers, bees found only a small portion of the presented flowers (as seen in both experimental studies this model is based on ; [13-22]). Any exploration segment has a chance of leading to no flower being found. However, the model does not replicate such behaviour, driving the bees to do more visits to different flowers from the first bout. Finding less flowers in the beginning of their flights leads to more repetitions of similar transitions and thus a better similarity index for real bees, when compared to simulated ones. This is now explained in the Discussion (Lines 283-294). We changed Appendix S3 accordingly (see Supplementary Materials, Lines 354-355).

**Comment 1.7 :**

Lines 188-192 this decrease/increase in non-rewarding flower visits is accompanied by a corresponding decrease/increase in total number of flower visits, correct ? (and lines 208-211 for encounters on flowers)

> Yes for the number of visits to non-rewarding flowers. Text lines 216-219 now reads « These variations in exploitation and interference competiton occurences correlate to variations in the total number of visits to flowers, effectively improving the bees’ foraging efficiency. This effect is stronger for the exploitation competition as it is occurring much more often (exploitation: 2 to 10 occurrences in average; interference: 0 to 2 occurrences in average; Fig 3A-3B).».

**Comment 1.8 :**

Throughout the results, I kept missing the ‘and model 3’ in between all of the parenthetical GLMM results and getting confused that I had missed something. I don’t have a suggestion for a way to avoid this, but if you can think of one, I’m sure future readers would appreciate it !

> We agree. Following the suggestion from reviewer 2, we moved all statistical results in Table 1 (Lines 174-176) and Table 2 (Lines 267-271).

**Comment 1.9:**

Lines 395-400. Are flowers in the same patch really 20m apart? This seems quite far!

> Chosen distances reflected the experimental data the model was tested against [13,22], but ultimately did not matter as only relative distances were used. We now address this question in text lines 426-430, which now reads « The distances used in the simulated environments were chosen to replicate the experimental data used to test the model [13,22], where closest flowers were spaced by 25 metres. However, in our model, only the relative distance between the different elements of the environment mattered as all distances were normalized in the process of creating the probability matrix (Appendix S1). ».

In addition, we now define these artificial flowers more clearly in the text (Lines 414-416) : « The simulated flowers should thus be considered as feeding sites such as plants or inflorescences, which are more likely to contain such large amounts of resources (20% of the bee’s crop) ».

**Comment 1.10:**

How realistic is it to use exactly the number of flowers for the bees to fill their crops? Does this have an affect on their behaviour?

> We now make it clear that the flowers should be considered as discrete feeding sites (inflorescencs or plants), see lines 414-416. We kept the terminology ‘flowers’ to evoke the artificial flowers used in experimental studies of traplining.

The question of the availability of flowers has been treated in Appendix S4 (with associated Figure S7 and Supplementary Video S1) where 5 bees were set to forage on environments of 20 to 100 flowers. A more detailed conclusion has been added to this appendix. The text (Supplementary Materials, Appendix S4, Lines 367-370) now reads : « As the number of flowers increases, positive reinforcement became more prevalent in driving partitioning, while negative reinforcement became less relevant. This result simply reflects how unlikely it becomes to come across competition as the resources become more available. ».

**Comment 1.11:**

The model implies that the bees are able to remember the locations and distances of all of the flowers in their environment. How realistic is this?

> The initial movement probability matrix is not meant to represent bees knowing their environment. The probabilities are approximations of the probability a bee would have to find each flower using a random walk. We added a new Appendix S6 to address this question (Supplementary Materials, Lines 412-439), but also added a sentence to explain it in the main text (Lines 443-445).

However, once flowers have been discovered, there is a strong hypothesis behind the way bees memorize their location. We judge this hypothesis to be reasonable because we used only a few flowers and bees are known to be able to learn few independant feeding sites and even make shortcuts between them (See references 66-67 in our paper). This statement has been added to the Methods (Lines 461-465).

**Comment 1.12:**

Line 428. This means probabilities greater than 1 can occur. Are any values greater than 1 that result from the multiplication by 1.5 set to 1?

> After any modification of a probability through the learning process in the model, all the probabilities of vectors departing from the same flowers as the modified vector were normalized to keep all probabilities under a value of 1 and their sum equal to 1. The text lines 467-469 has been changed to explain this more clearly.

**Comment 1.13:**

Line 437-438. Did you look at the effect on route similarity? Or any of the other metrics shown in figure 3? Although the focus of the paper is on resource partitioning, I would be interested to hear about the effects on route similarity as well as resource partitioning (and maybe the rest could be included in the supporting information).

> We now treat route similarity in the sensitivity analysis. The main text (Line 479) and the supplementary materials (Supplementary Materials, Lines 332-336 ; Figure S5) now show details of this analysis.

**Comment 1.14:**

Figure 1 appears to be missing an arrow and some yes/no statements for the ‘is the maximum flight distance reached?’ box.

> There was indeed some missing information. We revised the figure accordingly.

**Reviewer #2**

An individual-based model is presented that represents foraging of bumblebees and addresses the question how bumblebees develop „traplines“, i.e. preferred sequences of flowers they tend to visit during a foraging bout. While models on trapline emergence exist for individual bees, the question how two (or more) bees develop non-overlapping traplines and thereby avoid competition and increase resource partitioning has not been addressed with simulations yet.

The settings of the model are based on experiments with individual foragers, i.e. artificial landscapes with five (or 15) flowers, which refill their nectar store after each foraging bout (i.e., all bees of the current bout returned to their nest).

The question addressed is interesting. It is part of the overall topic of optimal foraging and the role of learning and cognitive capacities involved. The manuscript is well written and well-organized. Overall I think this is an interesting study that provides some important new insights, in particular that both, negative and positive reinforcement learning, are required to explain the emergences of non-overlapping traplines.

> Thank you for the positive words.

I am not an expert in bees or foraging theory, but in individual-based modelling, my comments therefore reflect a modeller’s perspective.

I like the model, but of course there are always assumptions that can be debated. Here, a key assumption is that, effectively, the bees are „omniscient“, they know the position and distance to all flowers in the landscape via their distance matrix. Of course, this is not the case in reality but rather a technical proxy. In reality, bees have a perception range, which means they certainly cannot know the flowers in clumps of flowers hundreds of meters away.

I understand from reading the ODD description, that within flower patches for the positioning of flowers „perception range“ played a role, but I think the authors should explain the underlying rationale in more detail, in particular when it comes to the landscape with three flower patches. I guess the main argument for assuming the distance matrix is a good proxy is that by taking the distance to the current position into account the probability of finding that flower by chance is taken into account, but this is something that could be tested. Why, for example, did you use the square of the distances ? Did you try different exponents ?

Thus, the model description should explain and justify the distance matrix in more detail, for example by : presenting a landscape and the typical distance matrix of a bee, before and after learning ; plotting the matrix elements over distance to get a feeling of how much less likely it is to find other patches, etc.

I am not saying the distance matrix is a flawed design, it is actually quite elegant, but, as it goes with proxies, you easily get used to using it and forget asking about its implicit assumptions, which might, for certain configurations, be quite unrealistic.

Thank you. The distance matrix used to estimate transition probabilities does indeed come with its own hypotheses and limits. We clarified its use by adding more information about it in the Methods (Lines 443-445; Lines 461-465) but also more importantly by adding a new Appendix S6 (Lines 412-439) in which we justify the use of this method by comparing it to the outcome of a random walk in a clear example.

**Further comments:**

**Comment 2.1:**

- Line 142: „GLMM …“ – I find these lengthy references to statistical metrics not very interesting by themselves, but then there are more than 50 of them in the main text. Yes, it makes everything look more „scientific“, but I think it would be sufficient to include this information in a Table in the Supplement, it would make the main text easier to read.

> Good point. We moved the statistical results in Table 1 (Lines 174-176) and Table 2 (Lines 267-271) as suggested.

**Comment 2.2:**

- I read the main text before looking at the figures and was slightly challenged by distinguishing model 1, 2 and 3. Perhaps say „model 1+, model 2-, and model 3+-“ to indicate also within the text that they about positive, negative, and combined re-enforcement? Just an idea.

> We re-labelled models in the text and the figures as suggested.

**Comment 2.3:**

- Line 191: „bees tended to increase their visits to non-rewarding flowers“ – since it is difficult to develop intuitions about how the distance matrix and its change due to learning, actually work, one cannot develop any institution about the reason for this observation. Still, the authors should be able to give a hint WHY this is so.

> Text (Lines 192-198) now reads : « The increase of non-rewarding visits in environments with patchily distributed resources can be explained as follows. If bees start reinforcing flowers of a shared patch, they will become more likely to visit the same patch. Given the much larger distance between flowers of different patches than between flowers of the same patch, the probability to switch from one patch to the next (without the help of the negative reinforcement) is low, leading to bees flying between the empty flowers of a patch repeatedly. This process ultimately increases visits to empty flowers, but also occurrences of interferences between the two bees if they are both at the same patch. ».

**Comment 2.4:**

- Line 211: „increased their frequency“ – same as in previous comment.

> Text (Lines 214-215) now reads : « Again this is likely due to the absence of negative reinforcement, leading bees to be trapped in an empty patch. ».

**Comment 2.5:**

- I like the outline of possible routes for making the model more „realistic“, but one important element is missing: most plants have more than one flower, how would this change the story? (Not that much, I guess, but being not a bee expert, I don’t have a good intuition here).

> We designed the model to replicate studies that investigated bee movements using « artificial flowers » and make new prediction within this framework. We clarified Lines 414-416 that these « artificial flowers » in fact emulate distinct feeding sites like plants or inflorescences, rather than single flowers.

**Comment 2.6:**

- Line 358: „pushed away“ – in terms of modulating the distance matrix, there is no difference between exploitation and interference competition, but could there be a difference in reality, with direct encounters with other bees having a stronger impact?

> This is an interesting point. We added a sentence about this in the main text, which now reads (Lines 488-490) : « To our knowledge, there is no empirical data suggesting that bees would react differently to these types of competitive interactions. Therefore we made the parsimonious assumption that the effect was the same. ».

As far as we know, this question has been seldom studied. Exploitation competition may be a much stronger driver than interference for competitive exclusion, primarily because bees rarely encounter on flowers and more often find empty flowers (e.g. in Henry & Rodet, 2018 *Scientific reports*, *8*1:1-10).

**Comment 2.7:**

- Speaking of „movement vectors“ is slightly confusing, as you are actually not using vectors in the model the way they are use in other foraging models where animals remember the position of rewarding sites. I also would not speak of „vector navigation“ as this indicates that, like in the models I mentioned (e.g. van Moorter et al. 2009, Milles et al. 2020), in models using vector navigation vectors are actually added to determine the direction for the next movement step. In your model, there is not such navigation or movement.

> Mentions of « movement vectors » and « vector navigation » have been replaced by the word « transitions ». Additionally, the way our model approximates navigation using random walk is now detailed in Appendix S6.

**Supplement**

Overall, the ODD model description is fine, but here still a few comments:

**Comment 2.8:**

- „Purpose and patterns“ – you did not list the patterns used to claim that model output is realistic enough for the purpose of the model.

> We added a new paragraph to this end (Supplementary Materials, Lines 12-14).

**Comment 2.9:**

- Line 29 „Internal parameters“ – see my comments on the rationale underlying the distance matrix.

> We added a sentence to briefly explain the distance matrix, and point to the new Appendix which gives a more thorough explanation (Supplementary Materials, Lines 35-37).

**Comment 2.10:**

- Line 147 „Initialization“ – this is about initializing the model entities and their state variables, not about setting model parameter, which has been intermingled here as well.

> Thanks. We removed mentions to parameter initialization from this part.

**Comment 2.11:**

- Line 177 „Input data“ – this is common misunderstanding of the ODD element which has been reported on both in Grimm et al. 2010 and 2020: this section is about the input of environmental variables, for example via data files, not about user input! This section should be moved to the begin of the Submodels section. You don’t have any “Input DATA”.

> The Input Parameters have been moved to the submodel part (Supplementary Materials, Lines 174-238).

**Comment 2.12:**

- Line 306 – I understand that indFlowerOutcome is an auxiliary state variable of the bees with all elements set to 1 after each foraging bout?

> It is a state variable which monitors the outcomes of the vectors used by a bee (positive or negative). At the end of a foraging bout, only the cells corresponding to the vectors used during the bout will have a value different from 0. They are set to 1 if the vector had a positive outcome (rewarding) or -1 for a negative outcome (non-rewarding). This state variable has been added to the table of Entities, State Variables and Scales (Supplementary Materials, Line 23), and its description in the Learning submodel has been modified (Supplementary Materials, Lines 302-308).

References

- Milles, A., Dammhahn, M., & Grimm, V. (2020). Intraspecific trait variation in personality‐related movement behaviour promotes coexistence. Oikos, 129(10), 1441-1454.

- Van Moorter, B., Visscher, D., Benhamou, S., Börger, L., Boyce, M. S., & Gaillard, J. M. (2009). Memory keeps you at home: a mechanistic model for home range emergence. Oikos, 118(5), 641-652.
